# The effects of nurses' spiritual well-being and death awareness on end-of-life nursing attitudes in Korea: a cross-sectional study

**DOI:** 10.7586/jkbns.24.023

**Published:** 2024-11-19

**Authors:** Hyemin Kim, Seunghye Choi

**Affiliations:** 1 Department of Nursing, Na-Eun Hospital, Incheon, Korea; 2 College of Nursing, Gachon University, Incheon, Korea

**Keywords:** Spirituality, Terminal care, Nurses

## Abstract

**Purpose:**

This descriptive survey was conducted to identify the factors influencing nurses’ end-of-life nursing attitudes, particularly focusing on spiritual well-being and death awareness among nurses in internal medicine and surgical departments.

**Methods:**

In total, 176 nurses from five medical centers in Korea completed a structured questionnaire. Data were collected on nurses’ general characteristics, end-of-life nursing attitudes, spiritual well-being, and awareness of death. Multiple regression analysis was conducted to examine the factors affecting nurses’ end-of-life nursing attitudes.

**Results:**

A significant difference in end-of-life nursing attitudes was observed according to the nursing department. Nurses in the internal medicine sector (wards and intensive care units) demonstrated significantly better end-of-life nursing attitudes than those in the surgical sector (surgical intensive care units). Death awareness exhibited a significant positive association with end-of-life nursing attitudes. The factors influencing nurses’ end-of-life nursing attitudes included a higher level of death awareness, and working in an internal medicine ward as opposed to a surgical intensive care unit.

**Conclusion:**

Nurses working in internal medicine wards exhibited higher levels of end-of-life nursing attitudes, indicating the need to develop specialized training programs in end-of-life care for surgical departments. In addition, it is necessary to improve nurses’ death awareness.

## INTRODUCTION

Nurses caring for dying patients reportedly feel powerless owing to their inability to alter the course of their patients' deaths, fear of handling postmortem procedures, anxiety stemming from the connection between the patients’ death and their mortality, and a profound sense of unsettlement in their values [1]. According to the American Nurses Association, nurses are obligated to apply their knowledge and skills to end-of-life care and cooperate with other healthcare providers to help patients and their caregivers feel comfortable during end-of-life care [2]. An end-of-life care attitude is defined as the consistent provision of physical, emotional, social, and spiritual care to patients and their families as they approach the end of life [3]. Previous research has shown that factors associated with nurses' end-of-life care attitudes include death awareness [4], number of end-of-life care encounters, religion, end-of-life care education experience, and spiritual well-being scores [5].

Nurses with more positive death awareness exhibit more positive end-of-life nursing attitudes [6]. In addition, nurses who value religion are more likely to have a positive awareness of death [6]. The more positive a nurse's attitude, the higher their level of end-of-life care performance [7]. Nurses who recognize the significance of spiritual care are 4.7 times more likely to assess a patient's religious background and four times more likely to incorporate spiritual care into their practice than those who do not [8,9]. According to the previous study, the nurses’ spiritual well-being was important to those nurses, which also has implications for enhancing the delivery of spiritual care interventions [10]. This suggests the critical importance of providing training in spiritual care and ensuring that it is provided by healthcare staff. Even among Korean intensive care unit (ICU) nurses who frequently care for terminally ill patients, the level of spiritual care practice was found to be low [11]. Factors hindering nurses from engaging in spiritual care include feeling unprepared [12] and a lack of confidence due to inadequate spiritual nursing education [13]. In fact, it has been reported that there is a perception that spiritual nursing education is unnecessary in universities and medical institutions [14] and that the responsibility for spiritual healing of patients is limited to religious individuals [15].

In addition, it was reported that Korean nurses’ attitudes toward end-of-life care and its influencing factors differ depending on the department [6,16,17]. ICU nurses are frequently summoned into action before death awareness manifests fully, resulting in high levels of end-of-life care stress [6]. In comparison, ward nurses experience role overload owing to their heavy workload, compounded by the decision-making and technical aspects of caring for dying patients [16]. In particular, internal medicine patients are significantly older and have higher severity of patient illness scores than surgical patients [17]. Therefore, it is necessary to closely investigate whether end-of-life nursing attitudes of nurses differ according to the department.

It is known that among the elderly who die in South Korea, approximately 78.9% of all deaths occur in medical institutions [18]. Therefore, end-of-life nursing attitude of nurses could affect the quality of death of patients. End-of-life nursing attitudes may vary depending on the nursing environment, research examining the factors influencing these attitudes, such as spiritual well-being and death awareness, across different work units with varying patient characteristics is lacking. Therefore, it is necessary to investigate how spiritual well-being and death awareness affect end-of-life nursing attitudes toward patients in various work units. The purpose of this study was to identify the factors influencing nurses’ end-of-life nursing attitudes, particularly focusing on spiritual well-being and death awareness among nurses in internal medicine wards and both internal and surgical intensive care units.

## METHODS

### 1. Study design

This cross-sectional study aimed to investigate the factors affecting end-of-life nursing attitudes among nurses.

### 2. Participants

The participants were nurses from five hospitals. The five institutions consist of an adult medical and surgical ICU at a tertiary university hospital in Incheon, three internal medicine wards at general hospitals in Incheon, and an internal medicine ward at a general hospital in Gyeonggi, South Korea. Convenience sampling was used to recruit participants from those who voluntarily consented to participate. The inclusion criteria were nurses with experience in caring for patients from 48 hours before death until death within the past six months. The exclusion criteria included new nurses with less than six months of working experience, outpatient nurses, operating room nurses, and manager nurses who do not participate in end-of-life care. The sample size was calculated using G Power 3.1.9.2. 172 participants were required for a multiple regression when set a medium effect size (0.15) [19], α = .05, power (1-β) = .95, and number of predictors = 10. We recruited 200 participants based on a dropout rate of 14%.

### 3. Instruments

A structured questionnaire was used to collect data. The questionnaire consisted of 99 items on general characteristics (13 items), spiritual well-being (20 items), death awareness (36 items), and end-of-life nursing attitudes (30 items).

#### 1) Participants’ characteristics

The nurses’ age, sex, marital status, religion, position, education level, clinical experience, years of work at the current hospital, working department, job satisfaction, number of end-of-life care experiences, experience in end-of-life nursing training, and difficulties in performing end-of-life care were investigated. Job satisfaction was assessed using a 5- Likert scale.

#### 2) Spiritual well-being

Spiritual well-being was assessed using a tool developed to measure spiritual well-being [20], translated into Korean and validated [21]. This tool was designed to measure not only religious well-being in the relationship between God and the transcendent but also a state of existential spiritual well-being that represents meaning, purpose, and satisfaction in life unrelated to religion. This tool consists of two factors, religious well-being (10 items) and existential well-being (10 items) with a 5 Likert scale, with higher scores indicating better spiritual well-being. Cronbach’s alpha was .93 at the time of development [22], and .89 in this study.

#### 3) Death awareness

The view of life and death scale was used to assess death awareness among the nurses [23,24]. This tool consists of five factors: positive and negative perceptions of death, anxiety about death, interest in death, and willingness to respect life. It consists of 35 items rated on a 5-point Likert scale, with higher scores indicating better death awareness. Cronbach’s alpha was .79 at the time of development [24], and .76 in this study.

#### 4) End-of-life nursing attitude

To measure end-of-life nursing attitudes, a tool FATCOD (Frommelt End of Life Nursing Attitudes Toward Nursing Care of the Dying Scale [3], a tool whose validity and reliability have been verified in Korea [25], was used. This tool consists of 30 items, with 20 items on nurses’ attitudes toward patients and 10 items on nurses’ attitudes toward the families of dying patients. This consisted of a 4-point Likert scale, with higher scores indicating better end-of-life nursing attitudes. Cronbach’s alpha was .94 at the time of development [25], and .73 in this study.

### 4. Data collection

Data were collected between October 10, 2023, and December 31, 2023. After sending an official letter regarding data collection to the head of the hospital’s nursing department and obtaining institutional approval, we explained this to the head nurse of the relevant ward and distributed the questionnaire to nurses who provided informed written consent. The completed questionnaires were submitted to a designated location, and after the survey was completed, the researchers collected them in sealed envelopes.

### 5. Data analysis

The collected data were analyzed using SPSS Win 29.0.1.0 (IBM Corp., Armonk, NY, USA). Of the 200 participants, analysis was conducted on 176 were analyzed, excluding 24 (refused to respond = 2, new nurses with less than six months of work experience = 10, nurse managers who did not perform direct nursing = 11, and insincere respondent = 1).

The sociodemographic characteristics of the participants are summarized using frequencies, percentages, means, and standard deviations. One-way ANOVA and independent t-test were conducted to examine differences in end-of-life nursing attitudes according to participants’ sociodemographic characteristics. One-way ANOVA and independent *t*-test were also used to examine the differences in spiritual well-being, death awareness, and end-of-life nursing attitude according to working department (internal versus surgical department). Scheffe’s test was conducted for post-hoc analysis. Pearson’s correlation coefficient was used to determine the correlations between age, clinical experience, years of work at the current hospital, spiritual well-being, death awareness, and end-of-life nursing attitudes. A multiple regression analysis was conducted to examine the factors affecting nurses’ end-of-life nursing attitudes.

### 6. Ethical considerations

This study was approved by the Institutional Review Board of Gachon University (IRB No. 1044396-202307-HR-135-01) and was conducted in accordance with the Declaration of Helsinki. All participants were provided with a form explaining the background and purpose of the study, survey content, benefits of participation, confidentiality, storage, and destruction of data, consent to participate in the study, right to withdraw from the study, and information about the researcher. Thereafter, written consent was obtained and the participants were offered a small gift as a token of gratitude.

## RESULTS

### 1. Participants’ characteristics

The mean age of the participants was 28.87 ± 5.39 years, and 88.6% of them were female The highest level of education was a bachelor's degree (n = 148, 84.1%), and 55 participants were religious. The nurses were staff nurses (n = 149, 84.7%) and charge nurses (n = 27, 15.3%), and the most common response to job satisfaction was moderate (n = 83, 47.2%). The mean scores for end-of-life nursing attitude increased in the following order: surgical ICU (SICU), medical ICU (MICU), and internal medicine ward (Table 1).

### 2. Differences in end-of-life nursing attitudes according to general characteristics

End-of-life nursing attitudes according to general characteristics are summarized in Table 2. Nurses working at general hospitals (*t* = 3.55, *p* < .001), religious participants (*t* = 2.25, *p* = .013), and charge nurses (*t* = -2.22, *p* = .016) had better end-of-life nursing attitudes than others. The nurses with moderate job satisfaction had significantly lower in end-of-life nursing attitudes than nurses with dissatisfaction (*t* = 3.56, *p* = .030).

### 3. The Differences in end- of- life nursing attitude, spiritual well-being, and death awareness according to the nursing department

There was a significant difference between the three groups when the departments were divided into internal medicine wards, MICU, and SICU (F = 9.19, *p* < .001). Additionally, we reclassified the three groups into internal (internal medicine ward and MICU) and surgical departments to identify differences by nursing department. End-of-life nursing attitude was significantly better among nurses working in internal departments than among surgical department nurses (t = 4.29, *p* < .001). There were no significant differences in spiritual well-being and death awareness according to nursing department (Table 3).

### 4. The Correlations among the age, clinical experience, years of work at the current hospital, end- of- life nursing attitude, death awareness, and spiritual well-being

Death awareness was significantly and positively correlated with end-of-life nursing attitudes (r = .42, *p* < .001), spiritual well-being was not significantly correlated with end-of-life nursing attitudes. Death awareness was significantly positively correlated with spiritual well-being (r = .19, *p* = .011) (Table 4).

### 5. Multiple regression to end of life nursing attitude

Multiple regression analysis was conducted to identify factors affecting end-of-life nursing attitudes. Six variables that were significant in univariate analysis included in the regression analysis: death awareness, hospital, religion, current position, job satisfaction, work department. In the regression model, hospital was removed due to multicollinearity problems, and the final model was satisfied the assumptions of the regression model. The Durbin-Watson statistic was used to obtain an autocorrelation error value of 2.10; thus, no autocorrelation was detected in the model. Multicollinearity was verified using the tolerance and variance inflation factor (VIF) values, and the tolerance was 0.85~0.92, and VIF was 1.09~1.19. The higher the death awareness (*p* < .001) and the working department was an internal medicine ward rather than the SICU (*p* < .001) the end-of-life nursing attitude was significantly better. The adjusted determination coefficient of the model (Adj R2) was 0.26 (F = 9.63, *p* < .001) (Table 5).

## DISCUSSION

This descriptive survey study was conducted among nurses from medical and surgical ICU and internal medicine wards to identify the factors influenced by spiritual well-being and death awareness on end-of-life nursing attitudes. The mean scores for the end-of-life nursing attitudes of nurses in internal medicine wards, medical ICUs, and surgical ICUs were 2.75, 2.74, and 2.60, respectively. Previous studies have considered end-of-life nursing attitudes to be positive when the mean score was 2 or higher [3]. Therefore, the end-of-life nursing attitudes of the nurses who participated in this study were found to be positive regardless of work departments. However, these scores were lower than those of nurses working in ICUs reported in previous studies conducted in Korea using the same tool [19,26]. Previous research indicated that end-of-life nursing attitudes among ICU nurses ranged from 2.82 to 3.01 [4,19,26], while those among internal medicine nurses were reported as 3.04 [27].

The findings indicated variations in nurses' end-of-life nursing attitudes based on departmental assignments. End-of-life nursing attitudes were notably better among nurses in the internal medicine department when comparing the combination of internal medicine wards and medical ICUs with surgical departments and among ward nurses when comparing ICUs with wards. Hence, it is plausible that patient characteristics in internal medicine and surgical departments exerted more influence on nurses' end-of-life nursing attitudes than departmental characteristics, such as ICUs and wards. This is consistent with previous studies demonstrating that nurses in internal medicine wards exhibited a relatively high level of end-of-life nursing practice [28] and that internal medicine wards displayed a higher level of end-of-life nursing practice than surgical ICUs [6]. A previous study has reported that internal medicine patients are significantly older and have higher severity of patient illness scores than surgical patients [17]. Previous study has reported that differences in end-of-life nursing attitude by work department are due to differences in the frequency of experiences in end-of-nursing care [29]. However, in this presenting study, there was no significant difference between frequency of experiences in end-of-nursing care and end-of-life nursing attitude. Previous study has shown that end-of-life care stress has a significant impact on end-of-life nursing attitude [30]. Although this presenting study did not directly investigate the end-of-life care stress, this may have influenced the end-of-life nursing attitude. Given that ICUs deliver significant end-of-life nursing care owing to elevated severity and mortality rates, it has been reported that ICU experience significantly impacts ICU nurses' end-of-life nursing attitudes [26]. Therefore, further investigations involving the end-of-life care stress and end-of-life nursing attitude among nurses are required.

This study examined the relationships between nurses' spiritual well-being, death awareness, and end-of-life nursing attitudes. In regression analysis, spiritual well-being was not a significant factor affecting end-of-life nurse attitudes. However, this study found significant positive correlations between death awareness and end-of-life nursing attitudes as well as between spiritual well-being and death awareness. Previous studies have demonstrated a positive and significant relationship between death awareness and spiritual well-being [4]. Nurses who maintain a positive perception of death tend to exhibit higher levels of involvement in end-of-life care, possess more positive attitudes, and experience lower levels of anxiety about death [31]. Therefore, it is crucial for nurses caring for dying patients to gain a healthy understanding of death. To achieve this, it is imperative to develop end-of-life nursing programs aimed at enhancing nurses' attitudes toward end-of-life nursing and death awareness.

Although current position was not a significant factor in the regression analysis, the charge nurses had significantly higher end-of-life nursing attitudes than the staff nurses. Previous studies have also indicated that nurses in higher positions [6,27]. with advanced education [4], and with more than 10 years of experience [32] tend to have more positive end-of-life nursing attitudes. Therefore, it is crucial for charge nurses to actively mentor and guide general nurses with less than ten years of experience, encourage them to adopt supportive attitudes toward patients during end-of-life care and help them improve their end-of-life nursing attitudes.

In another previous study, it was found that nurses in internal medicine wards exhibited better end-of-life nursing attitudes compared to nurses in surgical ICUs, which aligns with our findings [28]. Additionally, in a previous study, a higher number of patient death experiences was associated with more positive end-of-life nursing attitudes [31]. However, in this study, the number of end-of-life nursing experiences did not differ significantly according to the work unit. This suggests that positive end-of-life nursing attitudes may not develop naturally as the number of end-of-life nursing experiences increases. Therefore, further research on end-of-life nursing programs is warranted to promote positive perceptions of death and enhance end-of-life nursing attitudes in units with low awareness of death.

Organized end-of-life nursing has been shown to alleviate stress and negative emotions, such as anxiety and depression, regarding patient mortality, while also enhancing the quality of death for dying patients [33]. Therefore, enhancing nurses' attitudes and performance in end-of-life nursing is crucial. In this study, the most common challenges in providing end-of-life nursing care, as reported through multiple responses, were lack of communication (61 responses, 25.8%), followed by lack of time (60 responses, 25.4%), lack of expertise (53 responses, 22.5%), lack of specialized skills (38 responses, 16.1%), and fear of death (16 responses, 6.8%). Previous research has highlighted nurses' feelings of powerlessness and fear of handling post-mortem procedures when caring for dying patients [1]. However, this study contradicts this notion, as poor communication emerged as the most common difficulty in end-of-life care, surpassing fear of death. Further research is warranted to enhance communication skills in end-of-life care, as effective communication during this period positively impacts the quality of life of patients and their families [34] and helps prevent depression and complicated grief in bereaved families. Poor communication can exacerbate challenges stemming from a lack of time; thus, additional research is needed to develop and implement communication protocols aimed at assisting nurses in managing time constraints and providing emotional support to dying patients and their families.

The limitations of this study were as follows. First, the study was conducted among nurses at a tertiary care hospital and general hospitals within two metropolitan cities, thereby restricting the generalizability of the findings. Replication of this study among nurses in various regions and hospitals of varying sizes is warranted. Second, this study included medical ICU, surgical ICUs, and internal medicine wards, but did not include other wards that frequently encounter dying patients, such as oncology wards, hospice wards, and surgical wards. Therefore, further research is necessary to discern the differences in end-of-life nursing attitudes across various departments. Third, in contrast to the findings of a previous study, nurses' spiritual well-being did not show a significant correlation with end-of-life nursing attitudes, contrary to the findings of previous study [35]. Therefore, it is need to replicate this study to explore these relationships further. Finally, end-of-life is one of the most sensitive events in patients’ and family members, and end-of-life care competencies are essential for nurses. Moreover, further research should comprehensively investigate strategies to improve nurses’ end-of-life competency, including knowledge of physiological indicators of dying people and corresponding nursing intervention.

## CONCLUSION

Factors influencing the end-of-life nursing attitude were the death awareness and the working department. Nurses working in surgical intensive care units exhibited poorer end-of-life nursing attitudes, indicating the need to develop specialized training programs in end-of-life care for these departments. In addition, it is necessary to improve nurses’ death awareness.

## Figures and Tables

**Table 1.** General Characteristics of Participants (N = 176)

Characteristics	Categories	M ± SD	n (%)
Age (yr)	20~29	28.87 ± 5.39	118 (67.0)
	30~39		45 (25.6)
	40~49		12 (6.8)
	50~59		1 (0.6)
Sex	Men		20 (11.4)
	Women		156 (88.6)
Hospital	General hospital		84 (47.7)
	Tertiary university hospital		92 (52.3)
Marriage	Yes		40 (22.7)
	No		136 (77.3)
Education	Associate degree		20 (11.4)
	Bachelor's degree		148 (84.1)
	Master's degree		8 (4.5)
Religion	Yes		55 (31.2)
	No		121 (68.8)
Position	Staff nurse		149 (84.7)
	Charge nurse		27 (15.3)
Clinical experience (yr)	1~3	5.08 ± 4.53	76 (43.2)
	3~5		31 (17.6)
	5~10		46 (26.1)
	10~15		14 (8.0)
	15~20		7 (4.0)
	20~25		2 (1.1)
Years of work at the current hospital	Under 1	4.33 ± 2.70	32 (18.2)
	1~3		64 (36.4)
	3~5		30 (17.0)
	5~10		30 (17.0)
	10~15		11 (6.3)
	15~20		7 (4.0)
	20~25		2 (1.1)
Department	MICU		19 (10.8)
	SICU		73 (41.5)
	Internal medicine ward		84 (47.7)
Job satisfaction	Very satisfied		4 (2.3)
	Satisfied		76 (43.2)
	Moderate		83 (47.1)
	Dissatisfied		12 (6.8)
	Very dissatisfied		1 (0.6)
Number of end-of-life care experiences	Fewer than 5 times		31 (17.6)
	5 to 10 times		36 (20.5)
	11 to 15 times		24 (13.6)
	More than 15 times		85 (48.3)
End-of-life nursing training	Yes		92 (52.3)
	No		84 (47.7)
End-of-life care training site^†^	Undergraduate class		46 (42.6)
	Graduate school class		-
	Continuing education		21 (19.4)
	Hospital job training		30 (27.8)
	Hospice education		8 (7.4)
	Other		3 (2.8)
Reasons for difficulties in end-of-life care^†^	Lack of time		60 (25.4)
	Lack of professional knowledge		53 (22.5)
	Lack of professional skills		38 (16.1)
	Lack of communication methods		61 (25.8)
	Afraid of death itself		16 (6.8)
	Other		8 (3.4)
End-of-life nursing attitudes	MICU	2.74 ± 0.17	
	Internal medicine ward	2.60 ± 0.24	
	SICU	2.75 ± 0.22	

M = Mean; SD = Standard deviation; MICU = Medical intensive care unit; SICU = Surgical intensive care unit.

^†^Multiple responses.

**Table 2.** Differences in End-of-life Nursing Attitudes according to General Characteristics (N = 176)

Characteristics	Categories	End-of-life nursing attitude	
		M ± SD	*t*/F (*p*)
Hospital	General hospital	82.70 ± 6.63	3.55 (< .001)
	Tertiary university hospital	79.03 ± 7.04	
Sex	Men	81.20 ± 9.00	0.28 (.390)
	Women	80.73 ± 6.82	
Marriage	Yes	81.05 ± 6.51	-0.29 (.388)
	No	80.70 ± 7.25	
Education	Associate degree	80.15 ± 6.79	0.16 (.857)
	Bachelor's degree	80.81 ± 7.24	
	Master's degree	81.75 ± 4.77	
Religion	Yes	82.54 ± 6.39	2.25 (.013)
	None	79.98 ± 7.24	
Position	Staff nurse	80.26 ± 6.91	-2.22 (.016)
	Charge nurse	83.66 ± 7.39	
Job satisfaction	Satisfied or very satisfied^a^	80.92 ± 6.88	3.56 (.030)
	Moderate^b^	79.93 ± 6.85	b < c^†^
	Dissatisfied or very dissatisfied^c^	85.46 ± 8.27	
Number of end-of-life care experiences	Fewer than 5 times	81.87 ± 8.03	0.85 (.467)
	5 to 10 times	80.94 ± 6.67	
	11 to 15 times	82.00 ± 6.23	
	More than 16 times	79.97 ± 7.10	
End-of-life nursing training	Yes	80.70 ± 7.25	0.13 (.450)
	No	81.05 ± 6.52	

M = Mean; SD = Standard deviation.

^†^Scheffe’s test.

**Table 3.** The Differences in End-of-life Nursing Attitudes, Spiritual Well-being, and Death Awareness according to the Nursing Department (N = 176)

Nursing department (n)	End-of-life nursing attitudes		Spiritual well-being		Death awareness	
	M ± SD	*t* or F (*p*)	M ± SD	t or F (*p*)	M ± SD	t or F (*p*)
MICU^a^ (19)	82.26 ± 5.39	9.19 (< .001)	57.94 ± 14.79	2.59 (.078)	111.52 ± 10.41	1.03 (.360)
Internal medicine ward^b^ (84)	82.70 ± 6.63	b < c^†^	51.75 ± 13.56		109.33 ± 11.42	
SICU^c^ (73)	78.19 ± 7.21		50.13 ± 12.62		107.39 ± 13.53	
Internal department (103)	82.62 ± 6.39	4.29 (< .001)	52.89 ± 13.93	1.34 (.090)	109.73 ± 11.23	1.25 (.106)
Surgical department (73)	78.19 ± 7.21		50.13 ± 12.62		107.39 ± 13.53	

M = Mean; SD = Standard deviation; MICU = Medical intensive care unit; SICU = Surgical intensive care unit.

^†^Scheffe’s test.

**Table 4.** Correlations among Age, Clinical Experience, Years of Work at the Current Hospital, End-of-life Nursing Attitudes, Death Awareness, and Spiritual Well-being (N = 176)

Characteristics	Age	(1)	(2)	(3)	(4)	(5)
	r (*p*)					
Clinical experience (1)	.75 (< .001)	1	-	-	-	-
Years of work at the current hospital (2)	.67 (< .001)	.88 (< .001)	1	-	-	-
End-of-life nursing attitudes (3)	.12 (.105)	.14 (.068)	.14 (.058)	1	-	-
Death awareness (4)	.04 (.601)	.01 (.877)	.05 (.523)	.42 (< .001)	1	-
Spiritual well-being (5)	.12 (.102)	-.03 (.737)	-.04 (.617)	.13 (.075)	.19 (.011)	1

**Table 5.** Multiple Regression for End-of-life Nursing Attitudes (N = 176)

Variables	Categories		B	SE	*β*	*t*	*p*
End-of-life nursing attitudes	Constant		59.26	4.31		13.74	< .001
	Death awareness		0.21	0.04	.36	5.27	< .001
	Position	Charge nurse	2.70	1.39	.13	1.94	.054
	0 = Staff nurse^†^						
	Religion	Yes	0.63	1.09	.04	0.58	.566
	0 = No^†^						
	Job satisfaction	Moderate	-0.74	1.01	-.05	-0.73	.464
	0 = Satisfied or very satisfied^†^	Dissatisfied or very dissatisfied	3.24	1.88	.12	1.73	.086
	Department	MICU	-0.63	1.59	-.03	-0.39	.694
	0 = Internal medicine ward^† ^	SICU	-3.71	1.01	-.26	-3.68	< .001
Adjusted R² = .26, F = 9.63, *p *< .001, Durbin-Watson = 2.10							

SE = Standard error; MICU = Medical intensive care unit; SICU = Surgical intensive care unit.

^†^reference variable.

## Data Availability

The datasets generated and/or analyzed during the current study are not publicly available to protect the participants but are available from the corresponding author upon reasonable request.
